# The economic benefits of increasing breastfeeding rates in Spain

**DOI:** 10.1186/s13006-020-00277-w

**Published:** 2020-05-04

**Authors:** Juan Antonio Quesada, Ildefonso Méndez, Rocío Martín-Gil

**Affiliations:** 1 Financial Direction, Murcia Health Service, Murcia, Región de Murcia Spain; 2 Department of Applied Economics, Faculty of Business and Economics, University of Murcia, Murcia, Región de Murcia Spain; 3 Department of Anesthesia and Pain Management, Morales Meseguer General University Hospital, Murcia Health Service, Murcia, Región de Murcia Spain

**Keywords:** Breastfeeding benefits, Promoting breastfeeding, Cost–benefit analysis, Exclusive breastfeeding, Health services research

## Abstract

**Background:**

Interventions aimed at promoting breastfeeding rates are among the most effective possible health policies available, with an estimated return of US$35 per dollar invested. Indeed, some authors found that a 10% increase in exclusive breastfeeding rates in the first two years of life led to a reduction in treatment costs of US$312 million in the US, US$7.8 million in the UK, US$30 million in China, and US$1.8 million in Brazil. Among high-income countries, Spain stands out for its low breastfeeding rate.

**Methods:**

We calculated the savings that the Spanish National Health System would have benefited from had breastfeeding rates been higher in Spain, both from the time of hospital discharge and at 6 months postpartum. We followed the methods used in similar studies carried out in the US, Italy, Australia, the Netherlands, and the UK, to conservatively estimate these potential savings by considering only the lower thresholds in all our estimates. Here we approximated the benefits of having increased exclusive breastfeeding rates based on the lower incidence of infantile pathologies among exclusively breastfed infants. Robust evidence indicates that among breastfed infants there is a lower prevalence of otitis media, gastroenteritis, respiratory infections, and necrotising enterocolitis. We obtained the estimated monetary cost of these diseases by combining their prevalences with data about their economic costs for diagnosis-related groups.

**Results:**

The estimated effects we calculated imply that the Spanish National Health System could have saved more than €5.6 million for every percentage point increase in exclusive breastfeeding rates in Spain during 2014.

**Conclusions:**

Breastfeeding is essential both for the health of mothers and the health and development of newborns but is rarely considered as an economic issue and remains economically invisible. In addition to the improved wellbeing of mothers and their infants, breastfeeding can positively impact society as a whole and should therefore be better defined in public policies. Thus, strategies aimed at increasing exclusive breastfeeding rates would likely contribute to lowering the fiscal burden of the Spanish National Health System. Moreover, the magnitude of these potential benefits suggests that such policies would likely be socially cost–effective.

## Background

Interventions aimed at increasing breastfeeding rates are among the most effective possible health policies available, with an estimated social return of US$35 per dollar invested [1]. Empirical evidence suggests that lower breastfeeding rates lead to higher economic costs in terms of economic growth [2–4], work and productivity [5–7], and environmental losses [8, 9], as well as the social cost in terms of both infant and maternal mortality [10]. Higher breastfeeding rates lower healthcare economic costs because they reduce the prevalence of several pathologies among both breastfed infants and breastfeeding mothers. To this effect, several studies have estimated the economic costs to healthcare systems in different countries according to several criteria. The original currencies of the cited studies are here used in order to avoid any confusion different exchange rates may introduce.

In 1997, Drane [11] estimated a saving of US$6.2 million on treatments had exclusive breastfeeding (EBF) rates at 3 months increased to 80% in Australia. In 2001, Weimer [12] estimated a saving of US$3.6 billion to the US if breastfeeding rates had increased from levels recorded at the time (65% at hospital discharge and 29% at 6 months) to the levels recommended by the US Surgeon General (75 and 50%, respectively). They based these calculations on the likely reduction in healthcare system costs arising from a lower incidence of three childhood diseases in breastfed children: otitis media, gastroenteritis, and necrotising enterocolitis. In 2009, this study was updated by Bartick and Reinhold [13] to include all childhood diseases shown by the Agency for Healthcare Research and Quality to have a lower incidence among breastfed infants: necrotising enterocolitis, otitis media, gastroenteritis, hospitalisation for respiratory infection, atopic dermatitis, sudden infant death syndrome, childhood asthma, childhood leukaemia, type 1 diabetes, and childhood obesity. These results showed that had 90% of babies been breastfed for the first six months of their lives, the US would have saved US$13 billion annually and 911 lives per year.

Cattaneo et al. [14] published a study in Italy which compared children exclusively or predominantly breastfed for the first three months of their lives to non-breastfed children or children who were both breastfed and who received non-breastmilk feeds during the same period. They estimated that an extra US$178 per child had been spent in treatment costs per year in the latter two groups. Similarly, in 2007, Buchner, Hoekstra, and Rossum [15] calculated that the Netherlands could have saved US$279 per child born if EBF rates at 6 months had increased to 100%. Indeed, in 2012 UNICEF UK published a report [16] entitled *Preventing disease and saving resources: the potential contribution of increasing breastfeeding rates in the UK*. They concluded that had the breastfeeding rates in the country at the time increased to 75% in neonatal units with a 45% EBF rate at 4 months, there would have been a reduction in the treatment costs derived from gastroenteritis (GBP3.6 million), respiratory infection (GBP6.7 million), otitis media (GBP750,000), and necrotising enterocolitis (GBP6 million), equating to a total of more than GBP17 million per year.

Indeed, Rollins et al. found that a 10% increase in EBF at 6 months or in breastfeeding rates at 1 or 2 years (depending on the country and pathology examined), would have led to a reduction in treatment costs of US$312 million in the US, US$7.8 million in the UK, US$30 million in China, and US$1.8 million in Brazil. The corresponding figures, had breastfeeding rates increased to 90% in the US, China, and Brazil, and to 45% in the UK, would have been US$2.5 million, US$223.6 million, US$6 million, and US$29.5 million, respectively [17]. A recent study in Indonesia [6] concluded that the healthcare system cost resulting from not breastfeeding was estimated at US$118 million annually, comprising US$88.6 million in provider costs and US$30 million in non-medical patient costs.

According to UNICEF [18], there is a negative correlation between ever-breastfed rates and economic development, estimated using the per-capita gross domestic product (GDP). While ever-breastfed rates in low and middle-income countries are well above 95%, the corresponding rates for high-income countries usually fall below 90%. Within the 19 highest income countries analysed, Spain stands out for its low ever-breastfed rate, which was only higher than France, Ireland, and the US. Other authors [19] explain the differences in breastfeeding rates among developed countries by analysing the effect of macro-level factors such as welfare state policies and public health initiatives in 18 high-income countries. They found that the most common pathway leading to high breastfeeding initiation rates was the combination of a high percentage of women in parliament, a low national caesarean section rate, and low family spending, high rates of maternity leave, or high rates of women working part-time. In turn, the most common pathway leading to low levels of breastfeeding initiation was a low national adherence to the Baby-Friendly Hospital Initiative.

The latter study [19] also suggested that the low ever-breastfed rates in Spain are the result of the shorter duration of maternity leave permissions, lower rates of part-time female employment, lower spending on family benefits as a percentage of the country’s GDP, and lower percentage of baby-friendly hospitals. The 2012 Spanish National Strategy for Sexual and Reproductive Health [20] only included some general recommendations to promote maternal breastfeeding, including training multidisciplinary health personnel, providing information to pregnant women, and increasing the percentage of baby-friendly hospitals. However, no well-known policy initiatives designed to increase other factors that determine breastfeeding initiation rates (mentioned above or otherwise) have been implemented in Spain since 2012.

Here we estimate the potential savings that the Spanish healthcare system could enjoy (resulting from the lower prevalence of some pathologies among breastfed infants) if breastfeeding rates had been higher in 2014, both at the time of hospital discharge and at 6 months postpartum. As in other similar studies [11–17], we took a conservative approach and focussed only on four pathologies for which there is robust evidence of a lower incidence among breastfed infants: otitis media, gastroenteritis, respiratory infections, and necrotising enterocolitis. This work provides a lower bound to the overall positive effect that higher EBF rates would have had in Spain which can be used in future cost–benefit analyses to inform policy decisions designed to encourage maternal breastfeeding.

## Methods

We aimed to estimate the benefits that increasing EBF rates (both at the time of hospital discharge and at 6 months postpartum) from their 2014 levels of 85 and 15%, respectively [21, 22] to 95 and 50%, respectively would have had. The latter target (50% EBF rate at 6 months) was established at the 56th World Health Assembly as an objective for the year 2020 [23]. Thus, we compared the number of children affected by each of these four pathologies in Spain and conservatively estimated how many would have otherwise been unaffected had EBF rates met these targets. We approximated these incidence rates using previously published estimates of the number of children affected by each of these diseases and the effect of maternal breastfeeding on their prevalence [10, 24–26].

We used the total number of children born in Spain in 2014 (426,303 children) according to the Spanish National Statistics Institute [27] and the data for the average costs for the diagnosis-related groups (DRGs) published by the Spanish Ministry of Health, Social Services and Equality for 2013 [28]. Because we only focused on the theoretical reduction in healthcare costs that would have resulted from a lower prevalence of these four pathologies, our estimates provide a lower bound for the overall economic return that would have likely been expected had EBF rates in Spain been higher.

## Results

### Otitis media

Otitis media (OM) affects infants and young children. It is estimated that half of children aged under 5 years old will suffer at least one episode of OM, with 30% of them presenting it on a recurring basis. According to the available evidence [24], the highest incidence (61%) of OM was observed between the first and fourth years of life and the incidence of OM among EBF children during their first six months of life was 26%. We combined these prevalence rates with the number of children born in Spain in 2014 and the breastfeeding rate in 2014 at 6 months of age (15%) to estimate that 237,663 cases of OM could have been expected in the first two years of life of children born in 2014; 93% of these cases came from artificially fed (AF) children. We calculated that there would have been only 185,441 cases of OM among children aged under two years if the EBF rate in Spain had been 50% at 6 months of age, with approximately 70% of these cases corresponding to AF children. That is, had the 6-month EBF rate increased from 15 to 50% among the children born in 2014, there would have been 52,222 fewer cases of OM during the first two years of these children’s lives.

As summarised in Table 1, we used the average cost of €2335 published for the DRG *70 otitis media and upper respiratory tract infections in people aged under 18 years* combined with the reduction in OM cases calculated above to estimate that by extending EBF to cover the first six months of life and increasing it to 50%, the annual medical costs for children born in 2014 would have been €97,536,293 lower. Therefore, a 1% increase in the EBF rate at 6 months of age would have reduced healthcare costs by €2.7 million in 2014 as a result of a lower incidence of OM during the first two years of life.

**Table 1:** Savings due to lower prevalence of otitis media associated with changes in exclusive breastfeeding

	Children born in 2014			€	€
EBF (%)	EBF	No EBF	Total Cases	Total Costs	Savings
**15**	63,945	362,358	208,036	485,690,930	
**40**	170,521	255,782	178,195	416,022,149	69,668,781
**50**	213,152	213,152	166,258	388,154,637	97,536,293
**70**	298,412	127,891	142,385	332,419,612	153,271,318
**100**	426,303	0	106,575	248,817,075	236,873,855

Note: we obtained the number of children born in 2014 in Spain from the Spanish National Statistical Institute data. In this table EBF refers to exclusive breastfeeding rates at hospital discharge. The prevalences of otitis media (OM) among EBF children and AF children were 0.25 and 0.53, respectively. The unitary cost was €2334.65

### Gastroenteritis

In 2014, the incidence of gastroenteritis (GE) in EBF children during the first six months of life was 14% compared to 31% among AF children. In agreement, another study [10] found that half of reported diarrhoea episodes were resolved with breastfeeding. By combining these prevalence rates with the number of children born in Spain in 2014 and the EBF rate at six months of life in the same year, we obtained a total of 121,283 cases of GE, with 92.6% of these corresponding to AF children (112,331 cases) and 7.4% corresponding to GE among EBF children (8952 cases). Conversely, only 95,918 cases of GE would have been expected for these children had the EBF rate during their first six months of life been 50%. In monetary terms, based on the price for the DRG *816 nonbacterial gastroenteritis and abdominal pain in minors aged under 18 years without complications* of €1963 in 2013, a reduction of 25,365 cases of GE would have reduced healthcare costs by €49,798,653 per year (Table 2). In other words, a 1% increase in the EBF rate at 6 months of age would have lowered healthcare costs by €1.4 million in Spain in 2014 by reducing the prevalence of GE.

**Table 2:** Savings due to lower prevalence of gastroenteritis associated with changes in exclusive breastfeeding

	Children born in 2014			€	€
EBF (%)	EBF	No EBF	Total Cases	Total costs	Savings
**15**	63,945	362,358	121,283	238,112,888	
**40**	170,521	255,782	103,165	202,542,421	35,570,467
**50**	213,152	213,152	95,918	188,314,235	49,798,653
**70**	298,412	127,891	81,424	159,857,861	78,255,026
**100**	426,303	0	59,682	117,173,302	120,939,586

Note: we obtained the number of children born in 2014 in Spain from the Spanish National Statistical Institute data. In this table EBF refers to exclusive breastfeeding during the first six months of life. The prevalences of gastroenteritis (GE) among EBF children and AF children were 0.14 and 0.31, respectively. The unitary cost was €1963.28

### Respiratory infection

In 2014, the prevalence of respiratory infection (RI) was significantly lower among EBF babies during their first six months of life than in AF infants [10, 25]. There is evidence that a third of RI episodes could have been prevented by breastfeeding with an estimated 37% prevalence of RI for children fed with formulas versus 25% for those who were EBF [25]. By combining these prevalences with the current EBF rate for Spain in 2014 at 6 months postpartum, we calculated a total number of 15,986 cases of RI among babies born in Spain in 2014 who were EBF during their first six months of life and 134,072 cases among AF babies. The corresponding prevalences had the EBF rate been 50% in 2014 would have been 53,288 and 78,866, respectively. Thus, 17,904 fewer RI cases would have emerged among these infants had the EBF rate been 50%. According to data for DRG 775 *bronchitis and asthma in minors under aged 18 without complications*, the average cost of RI in 2013 was €2789. Thus, healthcare costs would have been €49,942,010 euros lower per year had the EBF rate increased from 15 to 50% among the babies born in 2014 (summarised in Table 3). In other words, for every percentage point increase in the EBF rate, healthcare costs per year would have been €1.4 million lower because of the reduced prevalence of RIs.

**Table 3:** Savings due to lower prevalence of respiratory infection associated with changes in exclusive breastfeeding

	Children born in 2014			€	€
EBF (%)	EBF	no EBF	Total cases	Total Costs	Saving
**15**	63,945	362,358	150,059	418,561,610	
**40**	170,521	255,782	137,270	382,888,746	35,672,865
**50**	213,152	213,152	132,154	368,619,600	49,942,010
**70**	298,412	127,891	121,923	340,081,308	78,480,302
**100**	426,303	0	106,576	297,273,871	121,287,739

Note: we obtained the number of children born in 2014 in Spain from the Spanish National Statistical Institute data. In this table EBF refers to exclusive breastfeeding during the first six months of life. The prevalences of respiratory infections (RIs) among EBF children and children fed with AF were 0.25 and 0.37, respectively. The unitary cost was €2789.32

### Necrotising enterocolitis

Necrotising enterocolitis (NE) is the most severe gastrointestinal pathology of those treated in neonatal intensive care units and is a significant cause of neonatal death and a leading cause of emergency surgery performed on newborns. A previous estimate [26] suggested that the prevalence of NE in underweight babies (weighing between 1500 and 2500 g at birth) exclusively fed with expressed breast milk (EBM) or EBF was 1%, while the incidence among AF underweight babies was 7%. Because NE generally occurs during children’s first month of life, we used breastfeeding rates in the hospital instead of those estimated for the first six months postpartum. The EBF rate at the time of hospital discharge was 85% in Spain [22]. Thus, we estimated the potential resulting saving had almost all (95%) the underweight babies born in Spain in 2014 been fed expressed breast milk or EBF.

To estimate the number of underweight babies born in Spain in 2014 we assumed that 3 to 5% of babies born in developed countries such as Spain are underweight, and thus used the lower bound of this range to provide a conservative estimate. As the average cost for the DRG *618 Newborn birth weight of 2000–2499 g, without significant surgery, with major problems* was €5990, we calculated estimated savings of €459,644 had the EBF rate at the time of hospital discharge been 95%, as summarised in Table 4. This means that every percentage point increase in the EBF rate would have likely lowered healthcare costs (by reducing the prevalence of NE) by €8111.

**Table 4:** Savings due to lower prevalence of necrotising enterocolitis associated with changes in expressed breast milk or exclusive breastfeeding

	Children born in 2014			€	€
EBF (%)	EBF	No EBF	Total Cases	Total Costs	Savings
**85**	10,871	1918	243	1,455,538	
**90**	11,510	1279	205	1,225,717	229,822
**95**	12,150	639	166	995,895	459,644
**98**	12,533	256	143	858,002	597,537
**100**	12,789	0	128	766,073	689,466

Note: we obtained the number of children born in 2014 in Spain from the Spanish National Statistical Institute data. In this table EBF refers to exclusive breastfeeding rates at hospital discharge . The prevalences of gastroenteritis necrotising enterocolitis (NE) among EBF children and AF children were 0.01 and 0.07, respectively. The unitary cost was €5990.05

### Combined results

Considering all four diseases in conjunction, we found that increasing EBF rates at the time of hospital discharge and at 6 months postpartum from their 2014 levels (85 and 15%, respectively) to 95 and 50%, respectively, would have saved the Spanish healthcare system at least €197 million per year. These estimated savings amount to approximately €464 per child born as a result of the lower prevalence of the four pathologies we analysed. This implies that the potential savings to the Spanish National Health System would have exceeded €5.6 million per percentage point increase in the EBF rates in 2014. The estimated overall effects are summarised in Table 5.

**Table 5:** Savings due to lower prevalence of four diseases associated with changes exclusive breastfeeding

2014, €	Otitis Media	Gastroenteritis	Necrotizing Enterocolitis	Respiratory Infections	Overall sum
**EBF (%)** **Six months / hospital discharge**					
**40 / 90**	69,668,780.93	35,570,466.54	229,822	35,672,865	141,141,934
**50 / 95**	97,536,293.30	49,798,653.15	459,643	49,942,010	197,736,601
**70 / 98**	153,271,318.04	78,255,026.38	597,537	78,480,302	310,604,183
**100 / 100**	236,873,855.15	120,939,586.23	689,466	121,287,739	479,790,646

Note: we obtained the number of children born in 2014 in Spain from the Spanish National Statistical Institute data. In this table EBF refers to exclusive breastfeeding during the first six months of life except for necrotizing enterocolitis, for which EBF refers to the prevalence at the times of hospital discharge

## Discussion

To the best of our knowledge, this the first study to estimate the benefits in terms of savings to the Spanish National Health System of higher breastfeeding rates in Spain based on estimating the decrease in the prevalence of some pathologies among EBF children. Following similar studies [11–17], we only considered the four pathologies for which the empirical evidence for their lower incidence among breastfed babies is strongest. Here, we estimated the effects of increasing EBF rates from their 2014 levels to the thresholds of 95% at hospital discharge and 50% at 6 months of life.

This paper belongs to a set of recent reports that estimate the benefits to national health systems of increasing EBF rates [11–17]. Previous estimates of the monetary benefit of increasing EBF rates differ depending on the number of pathologies, country, time period, and different medical and, more generally, healthcare structures considered in each territory. Thus, the reported estimated savings per child range from €252 to US$3140; our estimates lie somewhere between these values. Our estimates indicate that increasing EBF rates at the time of hospital discharge and at 6 months postpartum from their 2014 levels (85 and 15%, respectively) to 95 and 50%, respectively would have saved the Spanish healthcare system at least €197 million per year. This would have amounted to an approximate saving of €464 per child born in 2014, as a result of the lower prevalence of the four pathologies we analysed here. This implies that the potential savings to the Spanish National Health System would have exceeded €5.6 million per percentage point increase in EBF in 2014.

## Conclusions

Apart from lower mortality rates and prevalence of diseases other than the four analysed here, our estimates suggest that the Spanish National Health System could benefit from very substantial economic savings if EBF rates were increased. This is particularly important because empirical evidence [29, 30] suggests that initiatives to increase breastfeeding rates can be implemented at a low cost which would make them cost–effective in social and economic terms. Our estimates can be used in cost-effectiveness analyses to determine the social value of policies that directly or indirectly affect breastfeeding rates such as those designed to increase the availability of female part-time employment, public spending on family benefits, or the percentage of baby-friendly hospitals in Spain. However, it should also be noted that breastfeeding patterns are very socially complex and creating change is a difficult problem. For this reason, and given the objectives set by the WHO for attainable short-term changes, here we presented the potential savings per percentage point increase in the current EBF rates.

### Limitations

In this study we only considered the morbidity rates for the first and second year of life and ignored conditions like type 2 diabetes and obesity, as well as results for health issues affecting mothers. Moreover, we only analysed four pathologies for which there is robust scientific evidence of a lower prevalence among EBF children and we always applied the lower bound when quantifying costs. Furthermore, we have not considered in our study the savings of less paternal absenteeism as a result of the lower prevalence of some pathologies among children.

## Data Availability

The datasets supporting the conclusions of this article are available in the Instituto Nacional de Estadística (INE) repository, http://www.ine.es/dyngs/INEbase/es/operacion.htm?c=Estadistica_C&cid=1254736177007&menu=ultiDatos&idp=1254735573002, and in the Ministerio de Sanidad, Consumo y Bienestar Social repository, https://www.mscbs.gob.es/estadEstudios/estadisticas/inforRecopilaciones/anaDesarrolloGDR.htm .

## Abbreviations

EBF: Exclusive breastfeeding
GDP: Gross domestic product
DRG: Diagnosis-related groups
AF: Artificial feeding
OM: Otitis media
GE: Gastroenteritis
RI: Respiratory infection
NE: Necrotising enterocolitis
EBM: Expressed breast milk
